# A review of covetics – current understanding and future perspectives

**DOI:** 10.1039/d2na00500j

**Published:** 2022-11-24

**Authors:** Devyesh Rana, Kätchen Lachmayr, Steven Raymond Lustig

**Affiliations:** Department of Chemical Engineering, Northeastern University, Lustig Lab 360 Huntington Avenue Boston MA 02115 USA s.lustig@northeastern.edu

## Abstract

Covetics are a novel class of metal–carbon composites traditionally fabricated in an induction furnace with high power electrical current in the liquid metal–carbon mixture. The electrical current facilitates chemical conversion of carbon feedstock into graphene–metal crystalline structures. We explore the synthesis mechanism and hypothesize that carbon–metal species, rather than purely-carbon ions, are the reactant species driving the covetic reaction. Experimental mechanical and electrical property characterization in aluminum, silver, and copper covetics demonstrates improved tensile, hardness, and conductivity of covetic metals over pure metal controls. The literature proves that significantly improved material properties are possible with homogeneously distributed graphitic carbon in metal. High resolution transmission electron microscopy shows stripe, multidirectional, and alternating carbon–metal plane lattice structure nanocarbon patterns for aluminum, copper, and silver covetics, respectively, as well as high- and low-carbon concentration regions. Covetic Raman spectra and theoretical calculations indicate characteristic graphene signatures and the possibility of aluminum–graphene and silver–graphene bonding. This review consolidates the current literature and provides new avenues for research.

## Abstract 2

Graphene forms in liquid aluminum under ionizing current within a graphite crucible. Cations generated at the crucible anode, migrate through liquid aluminum, aggregate at the cathode, and form graphene–aluminum covetics.

## Graphical abstract

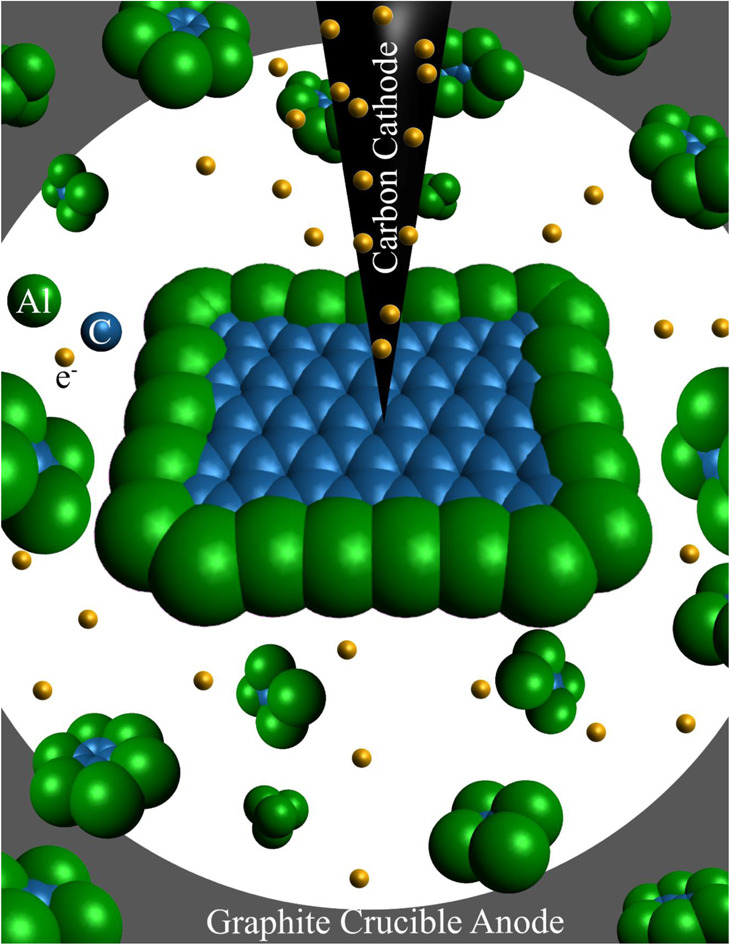

## 1. Introduction

There is an urgent need for next generation materials to advance infrastructures and electronics. The demand for metals is projected to increase 200% to 2 gigatons worldwide by 2050 for both new and ailing infrastructure.^1^ Key industries with increased demand include renewable energy, electric vehicles, electric power transmission, commercial and residential infrastructure, and strategic defense applications. It is critical to develop new materials that are lighter, stronger, more flexible, more conductive, or more corrosion resistant for longer lifetimes. Metal composites and alloys, at the forefront of current and future material usage, consist of a metal and one or more other materials, such as carbon.^2^ Modern carbon–metal composites demonstrate improved properties over pure metals, but there are several challenges to overcome. Some material property shortcomings are due to inhomogeneous nanocarbon distribution and incomplete carbon–metal bonding.

Covetics are a recently developed class of graphene–metal composite. Covetic materials exhibit lower densities than traditional alloys and metal composites,^3^ thereby having advantages in weight sensitive applications. Al-7075 covetics show improved ultimate tensile strength and yield strength, higher hardness, larger elongation, and lower density compared to Al-7075 base alloy.^4^ These properties highlight the potential advantages of covetics. However, covetics still face several challenges that prevent commercial application and large-scale production. There are neither standardized protocols, nor processes for synthesizing uniform and reproducible covetics. This results in conflicting experimental evidence of carbide formation during covetic synthesis. Difficulty in processing covetics leads to inhomogeneous distribution of carbon;^5^ which leads to low yields and undesirable properties. The governing chemical mechanisms, thermodynamics, and kinetics are unknown. We believe that a thorough understanding of these basic issues will enable scientists and engineers to overcome the process challenges, enabling commercial production and application.

This review summarizes the state-of-the-art knowledge of covetic materials. First, we describe the covetic synthesis and the hypothesized reaction mechanism. We offer mechanism refinement by describing the carbon feedstock degradation and graphene formation as an electrochemical reaction. We highlight the techniques for chemical characterizations and their findings. Although there are no systematic process-property studies published, we survey mechanical and electrical properties reported in recent publications. We then present quantum mechanical studies that assess the roles of relative metal atom size, chemical bonding, and graphene structures that form covetics. Finally, we conclude with future perspectives and opportunities for covetics. We hope this review will aid future researchers to identify new avenues to explore and advance the covetics field.

## 2. Synthesis

Several research groups developed different methodologies to synthesize covetic materials. The original patents by Shugart *et al.* and Scherer *et al.* outline basic processes for synthesizing Ag, Al, Au, Cu, Fe, Ni, Sn, and Zn covetics using various fabrication methodologies.^6–10^ The first covetics patent in 2010 describes the synthetic processes for copper covetic formation.^6^ A graphite crucible, which is electrical ground, is positioned in a gas heated or induction furnace to which copper is charged. Carbon, in various allotropes but specifically activated carbon or exfoliated graphite, is introduced into the molten copper. An arc welder carbon electrode is inserted into the mixture and a current is applied while mixing. The current range spans 135 to 240 A. Temperature shocks due to endothermic reactions are minimized with additional heat. An electrical current of 135 A is supplied using an arc welder carbon electrode, which is inserted into the molten mixture in example 1. The current is thought to draw the carbon into the copper actuating endothermic copper–carbon reactions. It was observed that the temperature drastically drops during the reaction from 1279 to 1082 °C. The arc welder supplied 230 A into the mixture in example 2. A similar temperature drop was observed to the first example and overcome by supplying additional heat. After cooling, the copper–carbon composition was remelted, and no phase separation was observed. Exfoliated graphite is used in example 3 as the carbon source with 240 A supplied for the reaction. After cooling, two distinct copper and carbon phases were observed where it was hypothesized that the copper–carbon composition was not formed. The endothermic product is theorized to result in a nano-composite material and only formed in the presence of an electric current. An exact chemical structure is not identified but is hypothesized to occur as a nanocomposite. This initial covetics work documents a synthesis process and potential product phases – copper–carbon, copper, and carbon.

The second patent in 2011 expands upon the works by Shugart *et al.*^6^ to synthesize gold, silver, tin, lead, and zinc covetics.^7^ Shugart *et al.* further refine the covetic synthesis process to include AC or DC currents for current application and that the current may be applied intermittently in periodic or non-periodic increments using an arc welder with a carbon electrode. The negative and positive electrodes are separated between 2–7 inches, which is believed to influence the bonding rate of the metal and carbon. This work also expands the metal criteria to include pure and alloy metals and carbon compositions from 0.01–70% by weight. Gold covetics were produced using an electric induction furnace mixing molten gold and activated carbon. A graphite electrode was attached to the welding rod to supply current to the mixture. The mixture temperature drops from 1093 to 926 °C suggesting an endothermic reaction, therefore additional heat was applied. After the reaction time, the mixture was cooled and was observed to be in a single gold–carbon phase with no visible phase separation. Additional testing of gold covetics also yields greater thermal conductivity and fracture toughness compared to unprocessed pure gold control. The same setup is used for silver, tin, lead, and zinc covetic synthesis with differing only the melting temperatures of 1015, 382, 287, and 478 °C, respectively. Exact structures are not identified; however, it is hypothesized that covetics are nanocomposite materials with single-, double-, and triple-bonds which do not break during re-melting.

The third patent in 2013 discusses the synthesis of aluminum covetics.^10^ An aluminum charged reaction vessel is heated to 871 °C. An agitator end of a rotary mixer is inserted into the molten aluminum and used to vortex the solution. Activated carbon is fed into the molten aluminum from 0.01 to 40% by weight. A carbon electrode affixed to a DC source is positioned into the grounded reaction vessel to provide a high current density during mixing. An arc welder intermittently supplies 315–379 Å through the mixture. After cooling there is a single aluminum–carbon phase. The aluminum–carbon phase is different from aluminum carbide, though the current chemical structure is unknown. Additional testing showed that the aluminum–carbon composition has greater thermal conductivity, fracture toughness, and ductility compared to traditional aluminum control.

The fourth patent in 2013 discusses the synthesis of iron covetics.^8^ An open-air reaction vessel is charged with iron. A graphite electrode is fixed to an arc welder and positioned into the vessel. The iron is heated and melted at an operating temperature of 1454 °C. An agitator end of a rotary mixer was inserted into the molten iron and vortexed. During mixing 378 A of direct current was applied intermittently and continuously. After cooling, the iron–carbon compound was observed to possess a single phase. No phase separation was observed upon remelting and solidification. The product structure is still unknown; however, further testing showed that the iron covetic had improved thermal conductivity, fracture toughness, and reduced grain structure compared to traditional iron control.

**Fig. 1:**
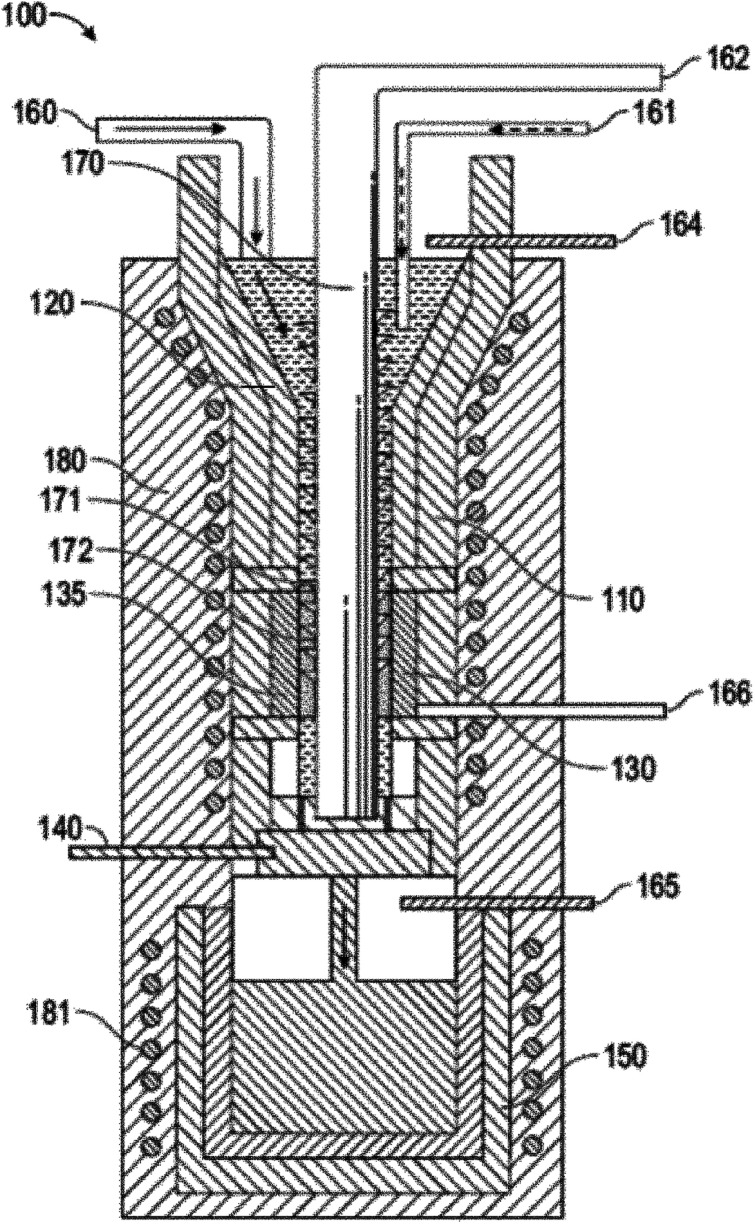
Cross-sectional view of a continuous reactor for producing composite materials, such as covetics by Scherer.^11^

Recently, a 2020 patent by Scherer *et al.* outlines a mechanism for continuous synthesis of covetic materials.^11^ The apparatus 100, shown in Fig. 1, includes an induction heated reservoir 110 having a first portion 120 and a second portion 130. A rotating auger 170 is arranged in the center of the reservoir 110 and traverses the first portion 120 and the second portion 130 of the reservoir 110. The rotating auger 170 includes threading 171 that extends from a shaft 172. The second portion 130 of the reservoir 110 hosts an electrical device 135 through which the rotating auger 170 passes. The apparatus 100 also includes a control valve 140 arranged between the second portion 130 of the reservoir 110 and an induction heated holding tank 150. The induction heated holding tank 150 includes an inert gas 165 (*e.g.*, argon). The apparatus 100 includes a first feed 160 for disposing a liquid metal into the first portion 120 of the reservoir 110, a second feed 161 for disposing a carbon material into the first portion 120 of the reservoir 110, a third feed 162 for providing a positive or negative electrical connection to the rotating auger 170, a fourth feed 164 for disposing an inert gas (*e.g.*, argon) into the first portion 120 of the reservoir 110, and a fifth feed 166 for providing a negative or positive electrical connection to the electrical device 135. The electrical device 135 may be negatively or positively charged by the fifth feed 166, while the rotating auger 170, which passes through a void defined by the electrical device 135, is positively or negatively charged *via* the third feed 162. The apparatus 100 also includes a first heating coil 180 that circumvents the reservoir 110, and a second heating coil 181 that circumvents the holding tank 150. This apparatus for continuous synthesis of covetic materials was applied to the synthesis of aluminum covetics to create rods, wires, and strips. No information regarding the product structure is given; however, testing showed that aluminum covetics had improved yield strength, ultimate tensile strength, and electrical conductivity is greater than pure aluminum controls. For example, the Vickers' hardness of aluminum 1350 (control, no additive, no covetic processing), aluminum 1350 (no additive; covetic processing), and aluminum 1350 covetic with 4 wt% graphene is 30.6, 30.1, and 33.0, respectively. The ultimate tensile strength of the same samples is 8.4, 8.2, and 8.6, respectively. The elongation at tear for the same samples is 40.4, 30.9, and 27.5%, respectively. This process equipment patent is the only covetic patent to give material property measurements, however, synthetic yields or mechanism details are not included in any of the patents.

**Fig. 2:**
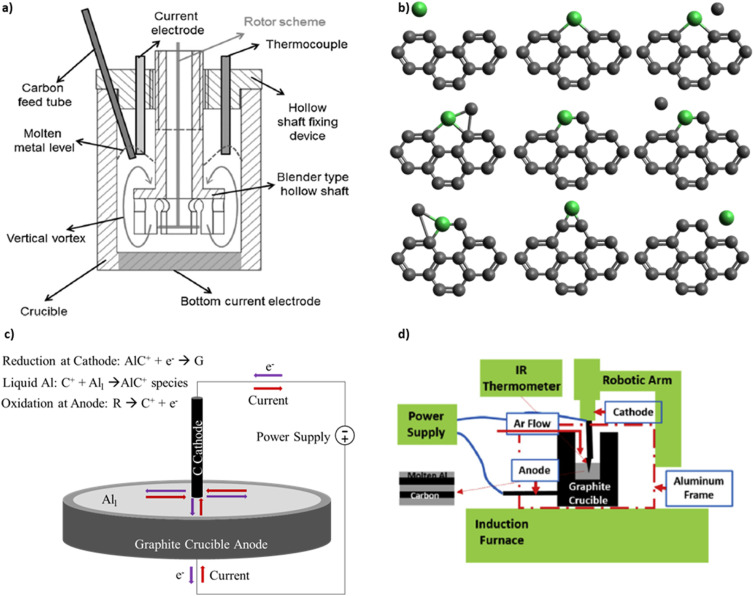
(a) Reactor schematic for covetic synthesis by Knych *et al.*^12^ (b) Metal catalysed graphene formation as outlined by Lee *et al.*^13^ (c) Representative schematic of aluminum covetic reaction using the model outlined by Levchenko *et al.*^14^ (d) Reactor schematic for covetic synthesis by Ge *et al.*^15^ (a) has been reproduced from ref. 12 under the CC BY license. (d) has been reproduced from ref. 15 with permission from Elsevier, copyright 2019.

Knych *et al.* published the first independent replication of the patent procedures and provide mechanical and electrical property measurements.^16^ The process setup by Knych *et al.*, shown in Fig. 2a, includes an induction furnace, a graphite crucible, an electrode with DC power supply, a stirring device, and an argon gas supply. The crucible has a 10 cm inner diameter and a 10 cm depth. The walls are electrically insulated so that current flows from the electrode centered at the top, through the liquid metal, and to the crucible bottom electrode. A lid seals the furnace to prevent oxide contamination from ambient air. This differs from the original patent designs that expose the liquid metal to ambient air. Copper and carbon nanotubes (CNTs) are placed into the crucible. The furnace is maintained at 1500 °C to allow copper to melt prior to electrical current application. Knych *et al.*^12^ measured a 0.6% decrease in density, 56% decrease in hardness, and a 56% decrease in electrical conductivity of copper covetics compared copper control. Secondary ion mass spectroscopy on the surface of sample slices detects carbon-rich and carbon-poor regions proving inhomogeneous carbon distribution. Kareem *et al.*^17^ fabricate aluminum covetics in an electric furnace, using an electric blender, graphite crucible, and four Solite® batteries as the DC power source. The graphite is rolled within aluminum foil and placed into the furnace at 850 °C. DC current application and mixing occur simultaneously. The molten mixture is then poured into a steel mold and air cooled. Kareem *et al.* measure a 0.186% decrease in density, 23.4% increase in hardness, and 43% increase in electrical conductivity of aluminum covetics compared with aluminum control. Kareem *et al.* note that non-uniform solidification creates kinetic dendritic aluminum structures within the aluminum covetics. Wang *et al.*^18^ use a floating catalytic chemical vapor deposition system to synthesize covetics. Silicon substrates are ultrasonically cleaned in ethanol and acetone and rinsed with deionized water. CNT films are tiled onto the silicon surface. An 800 nm thick copper layer is deposited onto the CNT surface using DC magnetron sputtering. The CNT/Cu composite is annealed in a vacuum annealing furnace. Wang *et al.*^18^ observe that copper grains cover the CNT, like a shell, after annealing. The copper shell increases the contact area between the copper and CNT and reduces the contact resistance. The reduced contact resistance between the copper and CNT promotes local ballistic conductor formation, where the number of local ballistic conductors formed is a function of the annealing temperature. However, no data are given for the relationship between the local ballistic conductors and either mechanical or electrical properties.

More recently, Ge *et al.*^15^ use an electrochemical reactor for laboratory-scale covetics synthesis. The reactor controls temperature, electrical power, and mixing simultaneously. An induction furnace is the main heat source for melting the metal. A graphite crucible serves as the anode. A robot-controlled graphite rod, that tapers to a point, serves as both cathode and mixing arm. The robotic arm spirals the cathode within the liquid aluminum for the purpose of homogenizing the carbon distribution. The crucible and induction coil are covered with a continuous argon flow to prevent aluminum oxidation from ambient air. Their induction furnace design is illustrated schematically in Fig. 2d. The reaction occurs for 10 minutes and ionizes 1.2 g of the activated carbon feedstock in the presence of an electric current. Simultaneously, the mixture temperature rises to 1100 °C from the initial melt temperature of 900 °C. The process starts by mixing 6061 aluminum and 5 wt% activated carbon. Electrical power is applied at 150 A and 10 V in a pulsed mode defined by repeating 2 minutes on and 1 minute off. The mixture temperature, voltage, current, and reaction volume are monitored.^15^ Ge *et al.* define the reaction volume as the liquid metal region which is exposed to current density greater than 100 Å cm^−2^ based on current distribution calculations using COMSOL. The COMSOL calculations model 100 Å cm^−2^ and 150 Å cm^−2^ electrical currents emanating from the graphite rod cathode and flowing throughout a three-dimensional solid aluminum block. The COMSOL model predicts a 1 cm^3^ region near the cathode tip with high current density. Beyond this region, the current density dissipates as current spreads throughout the aluminum block. After the reaction is stopped, the solution cools slowly under argon gas. Ge *et al.*^15^ hypothesize that a high current density is required for activated carbon conversion into covetic and that the reaction occurs mainly near the electrode tip. Ge *et al.* further hypothesize that the reaction only occurs upon reaching a critical carbon concentration. A detailed relationship between carbon concentration and covetic conversion remains to be investigated. High-resolution transmission electron micrographs indicate 5–100 nm carbon regions incorporated within the aluminum crystal lattice, referred to as lattice structure nanocarbon.^3^ Raman spectra of Al covetic cross-sections indicate an increase in graphene (G) to defect (D) peak ratio (G/D ratio). This very careful study clearly documents the synthesis process and product structure.

## 3. Reaction mechanism

Understanding the covetic reaction mechanism can ultimately lead to more efficient synthesis conditions, better reactors, and improved material properties. Ge *et al.* split the reaction mechanism for aluminum covetics into the following two steps: activated carbon degradation–ionization and carbon polymerization.^15^ In the first step, activated carbon feedstock degrades into carbon fragments that ionize under an electric current. Carbon and liquid metal mix from a carbon electrode that stirs the inside of a graphite crucible anode. A 10 V and 150 A DC application starts the covetic reaction, facilitates carbon–carbon bond breaking and reforming, increases atom migration, and promotes graphene formation. In the second step, carbon ions diffuse toward the high electric field density around the cathode tip. Upon reaching a critical carbon concentration, the carbon ions polymerize forming chains, ribbons, and finally graphene. The carbon ions react and bond with metal ions acting as nucleation sites for carbon nanoribbon growth and covetic formation. The mechanism is summarized as follows:^15^
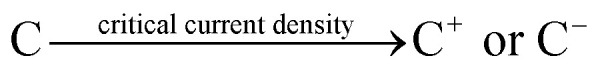
C^+/−^ + metal(Al) → M(Al) − C bondingC^+/−^ + C → C − C bonding or graphitic nano − structure

This covetics reaction mechanism is the latest hypothesis from the same research group built upon previous theories.^19–21^ However, molecular intermediates and kinetic pathways are still unknown. Therefore, an understanding of the reaction mechanism, including type of carbon ions from difference carbon sources, molecular weights, and species are needed. We acknowledge the significant challenge for obtaining molecular speciation, thermodynamic data, and reaction rates at high temperatures and in metals. Approaches that we are exploring in our lab are the use of computational methods, synthesis of room temperature melting point metal covetics, such as gallium, and the use of inelastic neutron spectroscopy. Results of these studies will be made available in future publications.

**Fig. 3:**
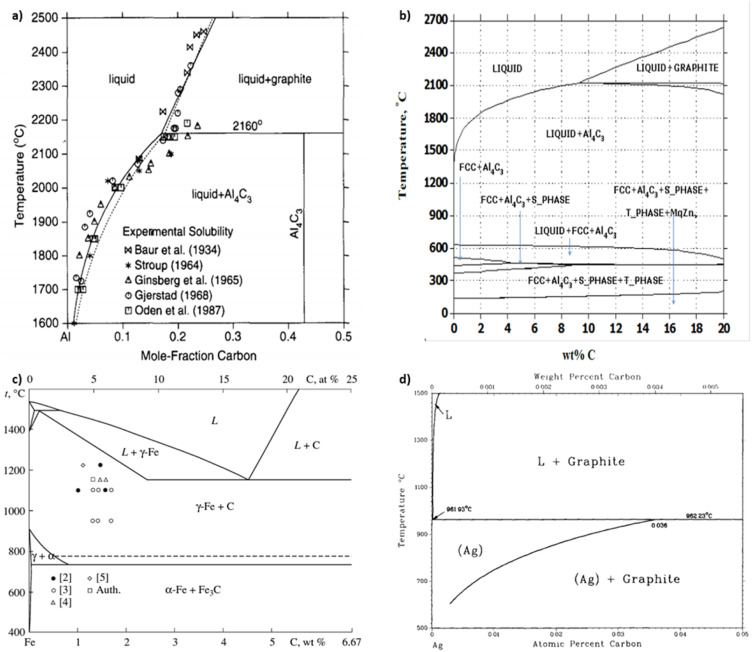
Aluminum–carbon phase diagram derived (a) experimentally from smelting^23^ and (b) computationally from CALPHAD.^5^ (c) Iron–carbon^24^ and (d) silver–carbon^25^ phase diagrams, (a) has been reproduced from ref. 23 with permission from Elsevier, copyright 1994, (b) has been reproduced from ref. 5 with permission from IDEALS, copyright 2014, (c) has been reproduced from ref. 24 with permission from Springer Nature, copyright 2010, (d) has been reproduced from ref. 25 with permission from Springer Nature, copyright 1988.

Liquid metal atoms can play a key role in carbon feedstock conversion into graphene. Iron, cobalt, and chromium catalyse hydrocarbon feedstock decomposition into carbon and hydrogen ions,^22^ and catalyse carbon ion polymerization into amorphous, filamentous, and graphitic carbon. Baker *et al.* fabricate filamentous carbon by acetylene decomposition in liquid iron, cobalt, and chromium.^22^ Baker *et al.* hypothesize that acetylene decomposition on the hot face of a gas reactor cell. Iron and cobalt are evaporated from a heated tungsten filament to combine with acetylene. The carbon and metal atoms migrate away from the hot face towards the cooler region, until carbon reacts with metal atoms. The carbon deposits polymerize into filamentous structures and terminate with metal atoms or clusters. As a result, carbon structures grow with random paths forming loops, spirals, and networks.^22^ Lee *et al.* hypothesize a more detailed nickel catalysed CNT growth mechanism, shown in Fig. 2b.^13^ CNTs form from hydrocarbon breaking into small graphitic structures. Nickel particles then bind to graphitic edges to form intermediate pentagonal structures. Additional carbon atoms further react with the nickel and graphitic edge rearranging into hexagonal rings, with the nickel. Finally, an additional carbon reactant replaces the nickel to extend the graphite structure. Finally, the nickel particles diffuse to other carbon clusters repeating the process as CNTs form. While evidence of nickel in the product suggests nickel diffusion, there is no observed or measured evidence of nickel diffusion. There are also no reports of CNT formation on aluminum. This may be due to a competing reaction forming aluminum carbide, illustrated in Fig. 3.

Large electric fields facilitate ion generation and diffusion during synthesis by directing cation migration from anode to cathode. The anode and cathode act as oxidation–reduction regions for hydrocarbon breakdown and graphene formation.^14^ Levchenko *et al.* produce graphene flakes in a high-current arc discharge apparatus with a carbon anode and cathode inside a vacuum chamber.^14^ Carbon and Y-Ni catalyst powder are placed in between the cathode and anode. Within the arc discharge, carbon oxidizes at the anode and enters the electric field generated plasma with the vaporized metal catalyst. The carbon and metal catalyst particles migrate from the anode towards the cathode. At the cathode, the metal condenses to form nanoparticles. Carbon particles aggregate around the metal nanoparticles and grow single layer graphene flakes (SLGF).^14^Fig. 2c depicts a simplified mechanism as it applies to covetics. At the anode, the hydrocarbon feedstock is broken down into ionic species. The cations migrate toward the cathode through the liquid aluminum to maintain an equilibrium. At the cathode, cations react with the electrons present at the cathode and form, with the aid of aluminum atom catalysts, into graphene and graphene–aluminum molecules.

Levchenko *et al.* hypothesize a magnetic field-enhanced arc assisted SLGF growth mechanism.^14^ First, an SLGF base layer nucleates and grows in radius, without additional layer formation. Upon reaching a critical size, a new graphene layer nucleates on top of the SLGF. Both layers continue to grow with additional nucleation centres forming at critical layer radii. The first layer determines the subsequent layer maximum radius. This process continues until the flake deposits onto the collection area. The main requirement for SLGF formation is low carbon density adsorbed onto a single graphene layer. The SLGF growth mechanism is comprised of four fluxes:

(i) *v*_dep_, carbon influx to the SLGF surface,

(ii) *v*_esc_, carbon outflux due to diffusion,

(iii) *v*_e_, frequency of carbon evaporation from the surface, and

(iv) *v*_C_, frequency of carbon ejection from gas molecule impact.

The SLGF forms when the carbon influx to the SLGF exceeds the carbon outflux for graphene layer nucleation and growth, *v*_dep_ = *v*_esc_ + *v*_e_ + *v*_C_. The study by Levchenko *et al.* suggests that carbon supply rate, ablation from the anode, and subsequent SLGF yield increases with increasing arc current.

We find plausible that aluminum covetic formation will follow a mechanism like the mechanism descriptions given by Ge *et al.*, Baker *et al.*, Lee *et al.*, and Levchenko *et al.* However, we hypothesize that molecular carbon–aluminum species, *e.g.* Al_4_C and Al_6_C_6_, rather than free carbon ions, are the main reactants in aluminum covetic formation. Within the liquid aluminum and in the presence of an electric field, graphene–aluminum species form from hydrocarbon breaking into low molecular weight carbon–aluminum species. Carbon–aluminum species aggregate and grow into aromatic carbon–aluminum species. Additional carbon–aluminum species further react with the aluminum–terminated graphitic edge extending the graphitic structures. Graphene increases in thermodynamic stability with increasing size.^30,31^ The graphene stability is due to the delocalized electrons within the aromatic carbon–carbon bonds. We speculate that within aluminum covetics it is possible that the liquid aluminum atoms may supply a thermodynamically stable termination group for the graphene resulting in larger graphene–aluminum. It remains possible, according to Ge *et al.*, that free carbon ions exist and are free to react to form graphene independent of any metal. *Ab initio* molecular dynamics studies presently conducted in our lab are providing more detailed insight. These studies will be available in an ulterior publication.

## 4. Carbides and oxides

Carbide is an important by-product to avoid in covetic production. As impurities within aluminum covetics, aluminum carbides are brittle and form defect points that weaken the bulk mechanical properties. In prior covetic syntheses, liquid aluminum (MP: 660 °C) and carbon are mixed at 900 °C.^15,17^ The final temperature reaches 1100 °C after DC application.^15^ X-ray diffraction, electron diffraction, and Raman spectroscopy highlight characteristic carbide phases within aluminum covetics, after casting and cooling.^5,15^ Experimental and theoretical phase diagrams indicate that aluminum carbide forms at temperatures below 2160 °C. The experimental phase diagram, in Fig. 3A, is derived from carbon solubility data from sodiothermic, electrolytic, and carbothermic smelting of aluminum for aluminum alloy synthesis.^23^ The theoretical phase diagram, in Fig. 3B, is calculated using the CALculation of PHAse Diagrams (CALPHAD) method. The CALPHAD method incorporates experimental thermodynamics to predict minimum temperatures and carbon concentration for various carbon–aluminum phases.^5^ A minimum temperature of 2160 °C and 0.15 mole-fraction carbon (experimental) or 2100 °C and 9 wt% carbon (CALPHAD) is needed to generate a two-phase graphite and liquid aluminum region. A carbide phase forms at temperatures under 2100 °C. Avoiding carbide formation requires exceeding a minimum temperature of 2100 °C with a minimum 8.7 wt% carbon during the reaction and then quenching the products into a solid phase before carbides can form during cooling. In the case of iron and carbon, a minimum temperature of 1200 °C and a minimum 4.6 wt% carbon is required for a single liquid iron and carbon phase to form ref. 24. Lower than 4.6 wt% carbon and higher temperatures than 1200 °C results in iron and carbon phase separation, shown in Fig. 3c. Similarly, a single liquid silver and graphite phase can be established at temperatures above 961 °C but at any carbon concentration, shown in Fig. 3d.^25^ A minimum carbon fraction could be the critical carbon concentration required for graphene formation within liquid metals, such as aluminum.

**Fig. 4:**
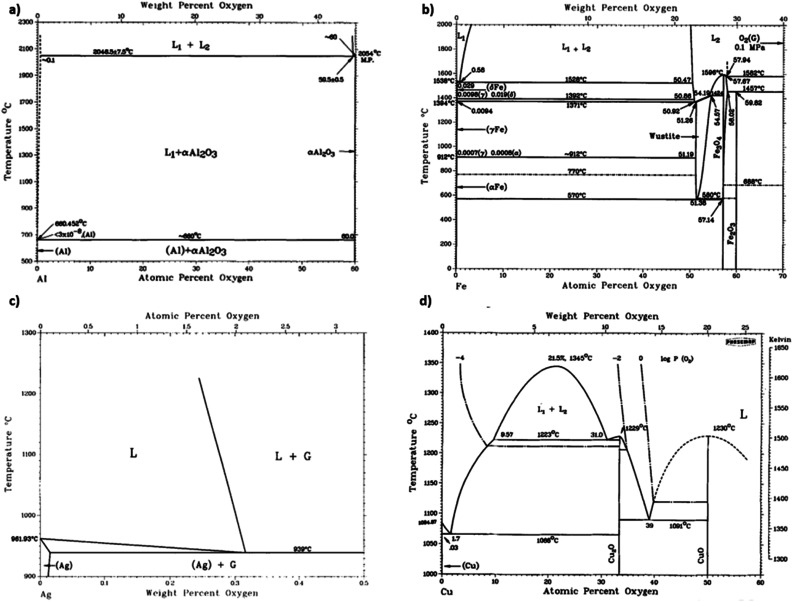
(a) Aluminum-oxide,^26^ (b) iron-oxide,^27^ (c) silver-oxide,^28^ and (d) copper-oxide^29^ phase diagrams, (a) has been reproduced from ref. 26 with permission from Springer Nature, copyright 1985, (b) has been reproduced from ref. 27 with permission from Springer Nature, copyright 1991, (c) has been reproduced from ref. 28 with permission from Springer Nature, copyright 1992, (d) has been reproduced from ref. 29 with permission from Springer Nature, copyright 2021.

Oxides are a significant contamination source for covetic synthesis. X-ray photoelectron spectroscopy spectra provide evidence of aluminum oxides on aluminum covetics with 3 wt% carbon.^20^ An aluminum oxide phase, shown in Fig. 4a, forms on aluminum in trace oxygen concentrations below 2046 °C.^26^ At temperatures above 2046 °C, the aluminum oxide forms a two-phase liquid aluminum – liquid aluminum oxide phase. There is no heating method to completely remove oxygen from aluminum oxide, therefore an oxygen free environment is required to avoid aluminum oxide formation. In aluminum welding, the alternating current tungsten inert gas process has been used to remove aluminum oxides through the mechanism known as the cleaning effect.^32^ The mechanism involves arc plasma anion generation in the direction of the aluminum base metal. The anion impact energy results in oxide bond breaking and removal.^33^ Iron oxides are rarely present in low oxygen containing environments with iron preferring αFe, γFe, or δFe phases.^27^ There needs to be at least an oxygen atomic percent of 51.38% for wurtzite formation, 54.57% for Fe_3_O_4_ formation, and 59.82% for Fe_2_O_3_ formation.^27^ Silver oxides readily phase separate into solid silver and O_2_ at temperatures above 190 °C under atmospheric pressure.^28^ However, as silver solidifies and cools from a melt, oxides invade the solid silver to form silver oxides.^28^ Oxygen solubility in copper is at a maximum at the eutectic temperature with a 0.03 atomic% of oxygen.^29^ Cuprite, paramelaconite, and tenorite can form at higher concentrations. A temperature of 1335 °C is required for phase separation between oxygen and copper. The electric current applied during covetic synthesis may be able to facilitate oxygen reduction, like the cleaning effect in aluminum welding.

## 5. Inhomogeneous carbon

Homogeneity and interfacial bonding between carbon and metal improve material properties. Aluminum alloys with uniformly dispersed CNTs have higher Rockwell hardness and tensile strength than pure aluminum, while CNT agglomeration and heterogeneity reduce these properties.^16,34^ Processes such as spark plasma sintering, chemical vapor deposition (CVD), induction melting,^35–37^ ball milling, and mechanical alloying improve CNT homogeneity within alloys. For example, carbon–aluminum composites synthesized by mixing aluminum and CNTs in a high energy planetary ball mill achieve five times greater hardness and seven times greater flexural strength than pure aluminum when smelt.^38^ To achieve this result Kwon *et al.* mixed aluminum powder and CNTs in a planetary ball mill for 3 hours under argon. After one week of passivation, the powder was transferred to a steel mold, under vacuum, and pressed at 750 °C for 1.5 hours. Kwon *et al.* claim that carbon homogeneously disperses within the metal matrix because of strong van der Waals forces.^38^ The flexural strength and yield strength of ball milled carbon–aluminum alloys and pure aluminum is 759 MPa and 108 MPa, respectively. In contrast, microstructure maps of aluminum covetics synthesized by Knych *et al.*, show varied carbon morphologies, phases, and presence from sample to sample.^12^ The three samples tested by Knych *et al.* contained low concentrations of clustered carbon, high concentrations of striated carbon patterns, and large concentrations of evenly distributed carbon. Precise carbon concentrations were not reported.^12^ TEM and Raman maps by Salamanca-Riba *et al.* reveal high, low, and zero carbon concentration regions.^3^ EDS map results from Brown *et al.* show converted and unconverted nanocarbon regions within covetic samples.^39^ There are varied Vicker's hardness's of covetic samples for different regions of these same samples. For example, the Vicker's hardness of an aluminum covetic disk, with 3 wt% carbon, at the center and 7 mm away from the center is 37HV30 and 42HV30, respectively.^39^ The results from Kwon *et al.*, with homogenous carbon distribution and 7× higher flexural strength, and Brown *et al.*, with inhomogeneous carbon distribution and varying hardness, highlights the necessity for homogenous graphitic carbon within covetics to achieve significantly improved material properties.

## 6. Properties of covetics

### 6.1. Mechanical properties

Experimental covetic densities approximately match theoretical densities that are based on the rule of mixtures.^4^ For example, the densities of Al-7075, amorphous carbon (particle size less than 100 nm, US Research Nanomaterials, Inc.), and graphene are 2.81, 1.80, and 2.27 g cm^−3^, respectively.^5^ Based on the rule of mixtures, the theoretical densities are 2.81, 2.79, and 2.77 g cm^−3^ for aluminum covetics with 0, 3, and 5 wt% amorphous carbon, respectively. Replacing amorphous carbon with graphene yields theoretical densities of 2.81, 2.79, and 2.78 g cm^−3^ for aluminum covetics with 0, 3, and 5 wt% graphene, respectively. Experimentally measured densities are 2.79 ± 0.22, 2.77 ± 0.19, and 2.76 ± 0.20 g cm^−3^, respectively, for Al-7075 covetics with 0, 3, and 5 wt% amorphous carbon.^4^ The density of pure copper is 8.90 g cm^−3^. Based on the rule of mixtures, the theoretical densities should be 8.90, 8.89, 8.89, and 8.88 g cm^−3^ for copper covetics with 0, 0.76, 0.83, and 1.96 wt% carbon. Average measured densities of six replicated copper sample measurements for each composition are 8.93, 8.93, 8.91, and 8.91 g cm^−3^, respectively.^12^ Incomplete carbon dissolution or carbide formation may result in decreasing density.^12^ Aluminum carbide density is 2.36 g cm^−3^, which lends support to this possibility. We hypothesize that greater carbide formation may lead to decreased densities and greater covetic formation may lead to theoretical densities, however, process limitations may need to be overcome to increase carbon retention and covetic conversion during synthesis.

**Fig. 5:**
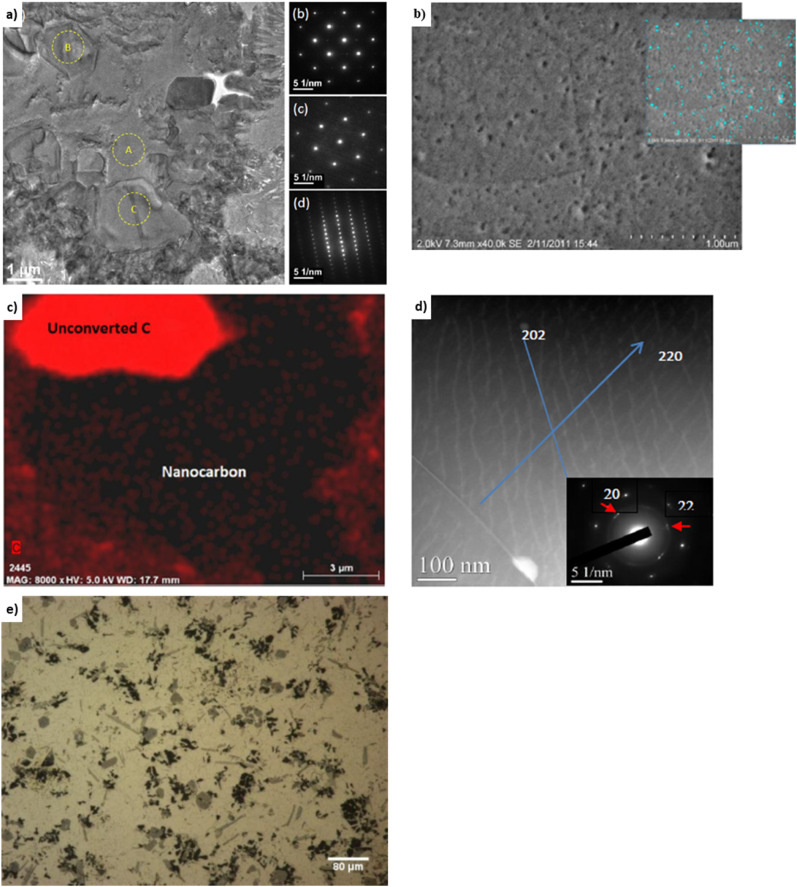
Representative (a) TEM,^43^ (b) SEM,^40^ (c) EDS,^40^ (d) high angle annular dark-field TEM,^19^ and (e) optical micrographs^20^ of Al-6061 covetic 3 wt% carbon, (a) has been reproduced from ref. 43 with permission from Springer Nature, copyright 2018, (b and c) have been reproduced from ref. 40 with permission from ASTM, copyright 2014, (d) has been reproduced from ref. 19 with permission from John Wiley and Sons, copyright 2015, (e) has been reproduced from ref. 20 with permission from IDEALS, copyright 2013.

Heat treatment affects strength, moduli, compressibility, hardness, and ductility for aluminum covetics. For example, the tensile strength of Al-6061 covetic with 3 wt% carbon is 29% greater than pure Al-6061.^39^ Brown *et al.* note the “puzzling” result of T6 treatment causing no significant difference in tensile strength between Al-6061 covetic and pure Al-6061, even though there is additional carbon within the covetic sample.^39^ The steps of heat treatment are heating, quenching, and aging. T6 heat treatment for aluminum heats the cast metal solution at 490 °C for 5 hours, quenches in cold water at 60 °C, and ages at 155 °C for 3 hours. T6 treatment increases grain sizes.^42^ A grain can grow larger by carbon atoms migrating from another grain or from a substitutional location to an interstitial location. Interstitial locations are between the metal lattice positions whereas substitutional locations replace a metal atom location with a carbon. Fig. 5a and b present aluminum covetic TEM and SEM micrographs in which aluminum crystallites of various orientations can be observed. Nanocarbon exists in 2.6% volume fraction.^39,43^ Area diffraction of regions “A,” “B,” and “C,” within Fig. 5a, indicate no overlapping grains and face-centered cubic aluminum along the [001] zone and rhombohedral-structured carbon–aluminum along [006] and [110] axes. The selected area diffraction regions indicate a preferential location for carbon atoms within the aluminum matrix. Fig. 5b highlights nanocarbon distribution, however, the energy dispersive X-ray of the C–K map, in Fig. 5c highlights both unconverted carbon and nanocarbon regions. The Al-6061 and Al-7075 covetic Young's moduli, both with 3 wt% carbon, are 17–29% and 37–38% greater than non-covetic counterparts, respectively.^20^ Aluminum covetic Young's modulus, shown in Table 1, and Rockwell hardness, shown in Table 2, are greater than their non-covetic counterparts. Overall, the Young's moduli of Al-6061 covetic with 2.3 wt% carbon, 3 wt% carbon, and 3.5 wt% carbon, are 28%,^19^ 17%,^3^ and 9%,^41^ greater than the control, respectively. In a parallel study, Kareem *et al.* fabricate Al-6061 covetics with 3 wt% carbon with a 20% increase in Young's modulus.^17^ The hardness of all covetics, shown in Table 2, is greater than their non-covetic controls. For example, the hardness of Al-6061 covetic with 2 and 3 wt% carbon is 111–375% greater than non-covetic. Brown *et al.* find varying Vicker's hardness along the radial direction with decreasing hardness toward the edges of the tested aluminum covetic sample. Fig. 5d, shows (202) and (220) diffraction patterns from graphite in aluminum covetics. The macroscopic carbon distribution within aluminum covetics is given in Fig. 5e, showing the overall non-uniform distribution of carbon.

**Table 1:** Young's modulus of various covetic studies as a percentage improvement from control metal. Other aluminum is used for unidentified aluminum source or grade

Metal	Carbon wt%				
	2	3	3.5	5	9
Al-6061	28 (ref. 19)	17,^3^ 20,^41^ 29 (ref. 39)	9 (ref. 17)		
Al-7075		37,^20^ 38 (ref. 4)		40,^4^ 41 (ref. 20)	
Other Al		18 (ref. 17)			
Cu		−3 (ref. 19)		0.01 (ref. 19)	−0.01 (ref. 19)

**Table 2:** Hardness in GPa for various covetic studies. Data estimated from given graphs or tables in references

Metal	Carbon wt%				
	0	2	3	5	9
Al-1359				1.1–1.5 (ref. 15)	
Al-6061	0.62 (ref. 19)	1.41 (ref. 19)	1.46–1.66 (ref. 39)	0.75 (ref. 15)	
			1.31–2.95 (ref. 17)		
Al-7075	2.00 (ref. 4)		2.25 (ref. 4)	2.75 (ref. 4)	
Cu	1.49 (ref. 19)	1.93 (ref. 12)	1.58 (ref. 19)	1.59 (ref. 19)	1.52 (ref. 19)

Copper covetics have not yet exhibited mechanical property improvements such as aluminum covetics. The Young's modulus values of copper covetics, shown in Table 1, are 3% less than pure copper.^19^ The Young's moduli of copper covetics with 5 to 9 wt% carbon are not significantly different.^19^ Hardness of copper covetics, shown in Table 2, with 2 wt% carbon is 29.5% greater than non-covetic copper. In a different study, hardness values of copper with 3, 5, and 9 wt% carbon are 6.0%, 6.7%, and 2.0% greater than non-covetic copper, respectively. Hardness increases towards outer sample regions, indicating a nonuniform carbon distribution. Liu *et al.* suggest that carbon clustering within materials widens the crystal lattice and increases lattice strain. Subsequently, this leads to weaker mechanical properties.^44^

Covetic materials show in-plane compression due to dissimilar graphene and metal thermal expansion coefficients.^45^ Aluminum and graphene thermal expansion coefficients are 23 × 10^−6^/°C and 4 × 10^−6^/°C, respectively.^46,47^ In our review, we were unable to find explicit experimental thermal expansion coefficient measurements, however, indirect observations are made related to thermal treatment. Bakir *et al.* observe that tensile strength and yield strength decrease upon annealing.^48^ This may be due to different graphene and aluminum thermal expansion coefficients. A mismatch in thermal expansion coefficients may break the bonds between the non-expanding graphene and the expanding metal ions during heat treatment. High strains may form resulting in lattice structure rearrangement within covetics. The high strain results in the weakening of hardness and tensile strength during heat treatment. Currently, covetics are air cooled due to the high synthesis temperatures involved; however, rapid quenching may reduce metal ion expansion and prevent any phase changes leading to thermal stress. However, more work needs to be done to understand the relationship between covetic thermal and mechanical properties during heat treatment.^48^

### 6.2. Electrical conductivity

Aluminum covetic electrical properties are directly affected by the electrical voltage and current applied during synthesis. For example, the electrical conductivities of aluminum covetic with 3 wt% carbon, pure aluminum, and graphene (calculated) are 38.1–67.3, 64.97, and 172% International Annealed Copper Standard (IACS), respectively.^52,53^ The electrical conductivity of bulk graphene has been reported to be 0.0034 to 1.38% IACS.^54–58^ Despite very high charge carrier mobility, graphene has low charge density resulting in low electrical conductivities. The electrical conductivities of aluminum covetic with 3 wt% carbon synthesized under 170 A/12 V and 170 A/24 V are 58.11% and 39.77% IACS, respectively. The electrical conductivities of aluminum covetic with 3 wt% carbon synthesized under 290 A/36 V and 400 A/36 V are 38.07% and 45.57% IACS, respectively.^17^ Aluminum covetic electrical conductivity increases with increasing voltage and/or current during synthesis.

Aluminum covetic electrical conductivity is affected by post-synthesis heat treatment. Table 3 summarizes electrical conductivities of aluminum covetic samples measured by local 4-point probe instrumentation. The electrical conductivity of Al-6061 covetic with 3 wt% carbon is 67.3% IACS.^59^ For the same sample, the electrical conductivity is 47.8% IACS after T6 heat treatment.^39,41^ This is similar to the conductivity of T6 heat treated parent aluminum.^60^ The electrical conductivity of TF, as fabricated with no heat treatment, aluminum covetic with 3 wt% carbon is 20% greater than the aluminum control. This same sample, after T6 heat treatment, shows no significant difference from the control.^39^ In comparison, the electrical conductivity of Al-6061 ranges from 37.6% to 48.5% IACS depending on heat treatment (TF, T6, T7).^60^ The heat treatment for T7 is thermally annealed for over 10 hours. The reduction in electrical conductivity after heat treatment is attributed to carbon and aluminum undergoing atmospheric oxidation during the heat treatment process resulting in aluminum oxide defects and carbon dioxide.^61^

**Table 3:** Electrical conductivity (% IACS) of aluminum, copper, and silver covetics. Covetics tested by Brown *et al.*,^39^ Isaacs *et al.*,^49^ Balachandran *et al.*,^50,51^ and Salamanca-Riba *et al.*^21^ are supplied by Third Millennium Metals, LLC

Metal	Carbon wt%	
	0	3
Al-6061	47.4 (ref. 39)	67.3 (ref. 39)
	24.5 (ref. 17)	38.1–58.1 (ref. 17)
Cu	101.4 (ref. 12)	100.2–102.4 (ref. 12)
	8.2 (ref. 18)	13.7 (ref. 18)
	30 (ref. 49)	30.5 (ref. 49)
	93.2 (ref. 51)	99.5 (ref. 51)
	100 (ref. 50)	87.9–93.3 (ref. 50)
Ag	106.9 (ref. 21)	96.9 (ref. 21)

Aluminum covetic electrical conductivity is proportional to carbon crystallite size and graphitic carbon concentration. Ge *et al.* fabricate aluminum covetics with increasing carbon crystallite size ranging from 0 to 45 nm yielding electrical conductivities ranging from 58 to 62 5 IACS, respectively. Aluminum covetics with carbon concentrations ranging from 0 to 3.6 wt% carbon yield electrical conductivities ranging from 58% IACS to 61.8% IACS linearly, as carbon concentration increases.^62^ It was observed that using 45 μm graphite particles with 200–400 nm carbon crystallite size, compared to 100 nm activated carbon crystallite particle size, resulted in a decrease in electrical conductivity. Sufficient carbon concentration is required for carbon rearrangement into graphitic nano-carbon with low enough crystallite sizes for high electrical conductivity.

The literature studies of electrical conductivities of copper and silver covetics report both increases and decreases in electrical conductivity compared to their non-covetic base materials. Pure copper and pure silver electrical conductivities are 100% and 108% IACS, respectively. Wang *et al.* synthesize copper covetics with 3 wt% carbon that exhibits an increase of 2.1% IACS compared to copper control.^18^ Their synthesis involves carbon–copper annealing on silicon substrates *via* magnetron sputtering. The procedure is different from other covetic synthesis methodologies, including a 600–700 °C operating temperature, which is below the copper melting point (1085 °C).^18^ Despite the obvious difference in the synthetic process by Wang *et al.*, they refer to their product as a covetic because of distinct copper and carbon (CNT) region formation. According to Wang *et al.* copper covetics have a (111) plane spacing of 0.244 nm which is like their experimental carbon–copper interface spacing. The Wang *et al.* findings may potentially expand the covetic definition to include other carbon allotrope formation beyond graphene. Isaacs *et al.* observe an 0.5% IACS increase for TMM provided copper covetics.^49^ Balachandran *et al.* observe a 7% increase in electrical conductivity for copper covetics, using copper ingots that are melted in a graphite crucible followed by graphite powder addition under a 200 mA current.^51^ In a following study, Balachandran *et al.* report a 7% increase in electrical conductivity for copper covetics with 3 wt% carbon.^50^ The drop in electrical conductivity is believed to be due to a structure of interfaces in the drawn wire covetic created by collapsed voids lined with carbon or due to iron impurities or due to small volume fraction of voids. The electrical conductivities of silver covetics with 5 wt% carbon and pure silver are 96.9% and 106.9% IACS, respectively.^21^ Further work is needed to have greater control and consistency in tuning electrical properties of copper covetics, which have reported increase and decrease in electrical conductivity, and silver covetics which have reported only decreasing electrical conductivity.

Crystallite, or grain, size is closely related to electrical properties of metals. Polycrystalline materials, such as metals, are composed of crystallites of varying sizes and orientations. Individual crystallites can be viewed as being made up of face-centered cubic or body-centered cubic metal atom clusters. A crystallite boundary is an interface between two crystallites in a polycrystalline material. Ge *et al.* find that a larger crystallite size correlates with higher electrical conductivity.^62^ Larger carbon crystallite sizes indicate an increase in the nano-crystalline graphitic network.^62^ Control of carbon and metal crystallite sizes will enable the control of covetic electrical conductivity.^21^

### 6.3. Grain & lattice structure

**Fig. 6:**
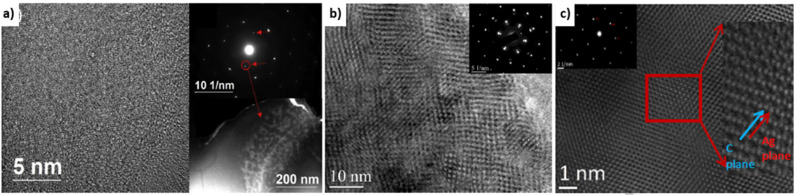
HRTEM Micrographs (a) aluminum,^15^ (b) copper,^3^ and (c) silver covetics and inset diffraction patterns showing graphitic weak spots.^3^ Aluminum covetic shows random modulation due to the presence of the graphite. Copper covetics shows modulation along with several crystalline directions. Silver covetics shows alternating planes between silver and graphene planes, (a) has been reproduced from ref. 15 with permission from Elsevier, copyright 2019, (b and c) have been reproduced from ref. 3 with permission from TechConnect, copyright 2012.

Covetic grain sizes can be significantly different from pure metal controls. Grain sizes of Al-6061 covetic with 3 wt% carbon and Al-6061 control are 1–30 μm and 100–200 μm in diameter, respectively, as measured by electron beam backscatter diffraction.^39^ Copper covetic grain sizes are similar to non-covetic copper.^3^ Grain sizes of Al-6061 covetic with 3 wt% carbon and Al-6061 control are 155.0 nm and 212.3 nm, respectively, as measured by XRD.^15^ The varying grain sizes observed within aluminum covetics may be caused by differing cooling rates after fabrication and other post-processing conditions resulting in two different carbon domains present within covetics: 50–200 nm size “particle nanocarbon” region (amorphous distribution), and 5–100 nm size lattice structure nanocarbon (LSNC) region (interconnected network).^15^ The LSNC for aluminum, copper, and silver are all different despite similar face-centered cubic arrangements, shown in Fig. 6. The LSNC for aluminum covetics is a stripe modulation in preferred crystallographic directions. The LSNC for copper covetics is multidirectional modulation in several crystalline directions.^3^ The LSNC for silver covetics is alternating planes between silver and graphene.

### 6.4. Chemical composition

Bulk carbon concentration measurements in covetic materials frequently yield inconsistent carbon concentrations. For example, carbon concentrations in Al-7075 covetic with 0, 3, and 5 wt% carbon loaded during synthesis result in 0.8, 2.2, and 4 wt% carbon, respectively, as measured by EDS.^4^ The carbon concentration of Al-6061 covetic with 5 wt% carbon loaded during synthesis measures 1.2 wt% carbon.^4^ The carbon concentration of copper covetic with 5 wt% carbon loaded during synthesis measures 3.5 wt% and 3.8 wt% carbon using XPS and EDS, respectively. Carbon concentrations for various copper covetics, consisting of less than 1% carbon loaded during synthesis measures 7692–20 000 ppm of carbon using secondary ion mass spectroscopy for repeated testing.^12^ There are many possibilities for varying carbon concentrations during measurement. EDS spectra indicate high and low carbon content regions consisting of both converted and unconverted carbon regions.^3,39^ Nanocarbon regions are also identified using high resolution transmission electron microscope, computed nano tomography, scanning electron microscopy, transmission electron microscopy, and X-ray transmission micrography, as shown in Fig. 5.^20,21,39,43^ EDS and XPS are problematic methods because low carbon concentrations and residual carbon within the microscope chamber can yield a “false positive” of carbon peaks.^12^ Glow discharge mass spectroscopy and secondary ion mass spectroscopy (SIMS) detect trace carbon amounts that correspond to unconverted carbon.^41^ SIMS shows low carbon content and uneven distributions.^12^ An analytical method that does not contain trace carbon content may yield valuable insight into the kinetics and reaction extents.

**Fig. 7:**
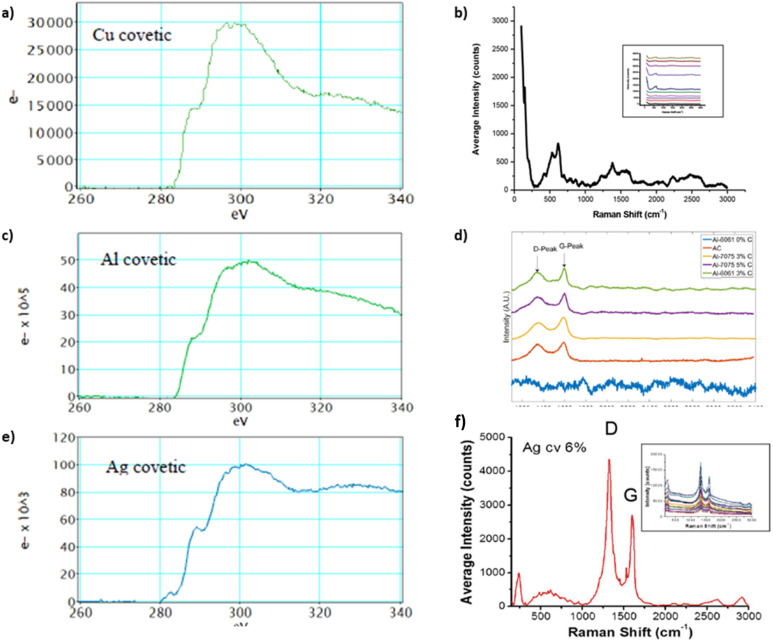
EELS and Raman spectra of (a and b) copper,^20,40^ (c and d) aluminum,^3,20^ and (e and f) silver covetics,^20,40^ (a, d), and (f) have been reproduced from ref. 20 with permission from Elsevier, copyright 2016, (c) has been reproduced from ref. 3 with permission from TechConnect, copyright 2012.

Raman spectra from covetic sample sections indicate the presence of characteristic sp^2^ and sp^3^ graphite signatures.^15,63^ In general, the G and D peaks are signature graphene and defect region vibrational modes, respectively, in the Raman spectrum corresponding to sp^2^ and sp^3^ bonding.^20,21,64^ Amorphous and activated carbon, used in covetic synthesis, are sp^2^ and sp^3^ bonded carbon allotropes without long-range crystalline order.^65^ Amorphous and activated carbon contain turbostratic allotropes that possess misaligned graphitic and amorphous carbon signatures. The electron energy loss spectroscopy graphs, shown in Fig. 7a, c, and e, of the striped carbon-rich regions in Fig. 6, show characteristic peaks related to sp^2^ and sp^3^ carbon regions also observed in amorphous carbon, activated carbon, CNTs, graphite, and graphene. The G peak, in Fig. 7, at 1580 cm^−1^ is from a primary in-plane vibrational mode and the D peak at 1350 cm^−1^ is the in-plane vibration resulting from the defect edges. A second-order overtone is observed at 2D near 2690 cm^−1^ with a 532 nm laser excitation. Regarding aluminum covetics, the D band shift is found at 1334 cm^−1^ in graphitic defects, but it is absent in pristine graphene. The G-peak shift at 1596 cm^−1^ shows a transition from amorphous activated carbon to nanocrystalline graphite under strain. Copper and silver covetic Raman spectra present similar graphitic carbon characteristic peaks.^3,21^ Aluminum, silver, and copper covetics Raman spectra indicate amorphous carbon signatures, presented in Fig. 7b, d, and f, between 1600–3000 cm^−1^.

## 7. Quantum mechanical modelling

**Fig. 8:**
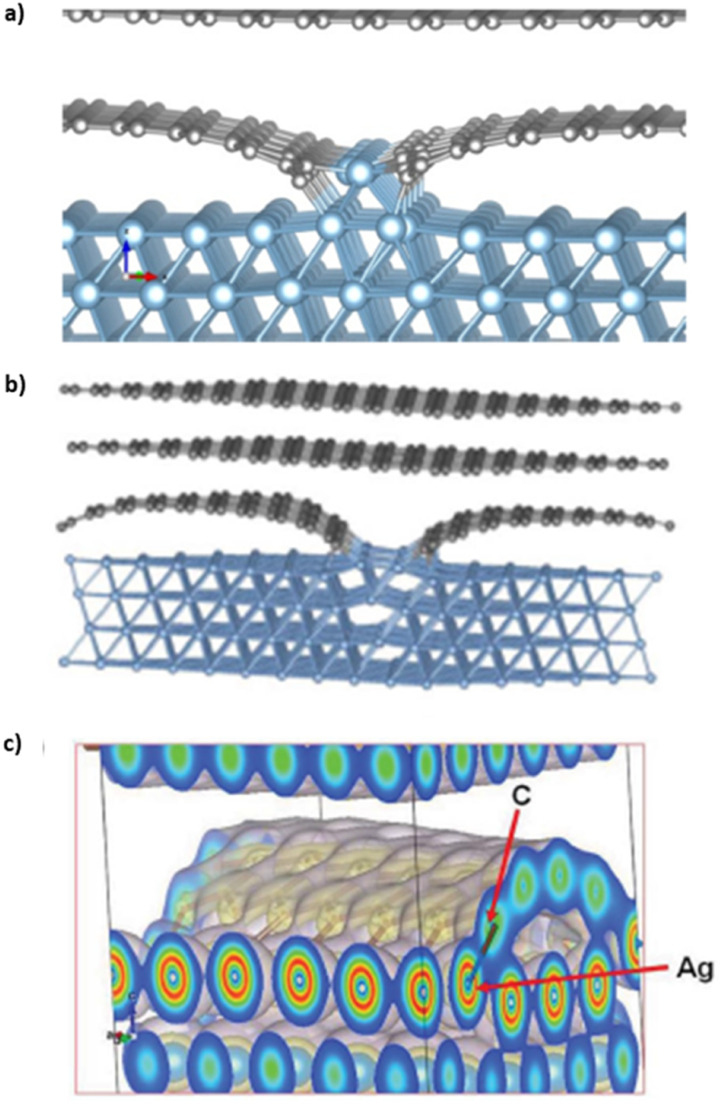
Optimized structure of (a) a solid graphene–aluminum covetic^20^ (b) and solid graphene–silver covetic.^21^ (c) Electron density spectrum showing the bonding between graphene and silver at the graphene edge,^21^ (a) has been reproduced from ref. 20 with permission from Elsevier, copyright 2016, (b) and (c) have been reproduced from ref. 21 with permission from Wiley, copyright 2015.

Quantum mechanical calculations on carbon–aluminum and carbon–silver models predict their electronic and geometric structures.^20,21^ Theoretical calculations on optimized carbon–aluminum models, shown in Fig. 8a, indicate that bonding between carbon and aluminum occurs at edges of ribbons and next to carbon vacancies. No bonding occurs between the aluminum block and carbon sheet in defect-free graphene models. The carbon and aluminum bonds have a covalent character. This is possible because of the similarities in the diatomic bond energies between Al–Al (2.78 eV) and Al–C, (2.74 eV).^20^ Similar theoretical calculations on optimized carbon–silver models are shown in Fig. 8b.^21^ Silver covetic vibrational frequencies indicate graphite modes at 127 and 1528 cm^−1^ relating sp^2^ bonded carbon atom vibrations and defect graphite modes at 1300 cm^−1^. Graphene sheet defects, such as vacancies and edges, are found between 300 and 1000 cm^−1^. Electron density graphs, in Fig. 8c, indicate that silver and carbon form common covalent electron orbitals.^21^ DFT calculations show that the bond between carbon and silver should yield peaks in the low energy range of carbon 1s and silver 3d, within XPS spectrum. However, no such peaks are observed *via* XPS. Salamanca-Riba *et al.* hypothesize that silver binds at graphene edge defects resulting in a low silver content bonding to carbon, relative to the number of carbon–carbon bonds.

## 8. Conclusions

Covetics are promising new metal composites. Some aluminum, copper, and silver covetics investigations report increases in tensile strength, hardness, and electrical conductivity compared to base controls. The electrical conductivities of copper and silver covetics still needs further investigation. The electrical conductivities reported for copper covetics are both better and worse than base copper controls, whereas for silver covetics the reported electrical conductivity is 9% less than base silver control. Some material property shortcomings are hypothesized to be due to inhomogeneous nanocarbon distribution and incomplete carbon–metal bonding. Improvements to synthesis understanding may lead to improved synthesis yields with consistent synthesis of mechanical and electrical properties.

There is a need to better understand different metal covetic syntheses, thermodynamics, kinetics, and specific reactants and products. Past publications do not demonstrate high carbon conversion, bulk carbon homogeneity, or high covetic yields. Chemical characterization techniques, such as Raman spectroscopy and electron energy loss spectroscopy, reveal that amorphous carbon converts into graphitic structures. However, both are surface measurement techniques without bulk material measurement capabilities. DFT calculations reveal bonding between graphene edges and aluminum and graphene edges and silver. However, more work is needed to validate theoretical models with experimental results.

Despite the current challenges, the potential for covetic materials to revolutionize current materials, with increased hardness or tensile strength or electrical conductivity or other mechanical or electrical properties, presents a remarkable opportunity. Increased mechanical properties, for example in steel, due to graphene–iron covalent bonding can provide the framework for next generation infrastructure materials. Increased electrical conductivity, for example in copper, can provide more efficient and higher throughput electrical components. We hope that this review helps researchers identify and address the challenges in covetics synthesis that remain a mystery such that commercial success is possible.

## Author contributions

Devyesh Rana: writing, Kätchen Lachmayr: reviewing and editing, steven Lustig: writing, reviewing, and editing.

## Conflicts of interest

There are no conflicts to declare.

## Supplementary Material
